# The Effects of Chemical and Mechanical Stresses on *Bacillus cereus* and *Pseudomonas fluorescens* Single- and Dual-Species Biofilm Removal

**DOI:** 10.3390/microorganisms9061174

**Published:** 2021-05-29

**Authors:** Inês B. Gomes, Madalena Lemos, Susana Fernandes, Anabela Borges, Lúcia C. Simões, Manuel Simões

**Affiliations:** 1 LEPABE, Department of Chemical Engineering, Faculty of Engineering, University of Porto, Rua Dr. Roberto Frias, s/n, 4200-465 Porto, Portugal; ibgomes@fe.up.pt (I.B.G.); deq11015@fe.up.pt (M.L.); up201304637@edu.fe.up.pt (S.F.); apborges@fe.up.pt (A.B.); 2 CEB-Centre of Biological Engineering, Campus de Gualtar, University of Minho, 4710-057 Braga, Portugal; luciachaves@deb.uminho.pt

**Keywords:** BDMDAC, disinfection, high-density polyethene, shear stress, rotating cylinder reactor

## Abstract

Biofilm control is mainly based on chemical disinfection, without a clear understanding of the role of the biocides and process conditions on biofilm removal. This study aims to understand the effects of a biocide (benzyldimethyldodecyl ammonium chloride—BDMDAC) and mechanical treatment (an increase of shear stress -$\tau_{w}$) on single- and dual-species biofilms formed by *Bacillus cereus* and *Pseudomonas fluorescens* on high-density polyethene (HDPE). BDMDAC effects were initially assessed on bacterial physicochemical properties and initial adhesion ability. Then, mature biofilms were formed on a rotating cylinder reactor (RCR) for 7 days to assess the effects of chemical and mechanical treatments, and the combination of both on biofilm removal. The results demonstrated that the initial adhesion does not predict the formation of mature biofilms. It was observed that the dual-species biofilms were the most susceptible to BDMDAC exposure. The exposure to increasing $\tau_{w}$ emphasised the mechanical stability of biofilms, as lower values of $\tau_{w}\;$(1.66 Pa) caused high biofilm erosion and higher $\tau_{w}$ values (17.7 Pa) seem to compress the remaining biofilm. In general, the combination of BDMDAC and the mechanical treatment was synergic in increasing biofilm removal. However, these were insufficient to cause total biofilm removal (100%; an average standard deviation of 11% for the method accuracy should be considered) from HDPE.

## 1. Introduction

Food-processing facilities have strict cleaning and disinfection standards to control spoilage and pathogenic microorganisms that can cause outbreaks of diseases and consequently constitute a risk for public health. This concern has a special focus when microorganisms adhere to a surface and form biofilms [1]. Biofilms are microbial communities adhering on surfaces and protected by a matrix of extracellular polymeric substances (EPS) produced by resident cells [2]. Current disinfection practices used in industrial settings apply clean-in-place (CIP) protocols. CIP aims to ensure high safety conditions for the correct operation of production lines. Frequently, CIP is based on the use of alkali detergents combined with biocidal compounds [3]. Moreover, acidic detergents are often applied to clean high food debris and mineral deposits. Furthermore, the hydrodynamic conditions also play an important role in CIP efficiency, as reviewed by Fernandes et al. [4]. The use of disinfectants and detergents is, sometimes, intercalated by rinses with water turbulence or scrubbing, to induce biofilm removal [5,6,7]. However, the low susceptibility and high viscoelastic properties of biofilms hinder their control [8].

The type of material used for industrial equipment and piping systems also influences the biofilm structure and behaviour, and specifically their tolerance to disinfection and cleaning procedures. The general principles for CIP circuits recommend the use of AISI316 or AISI304 stainless steel (SS) with an electropolished surface finish [9]. In addition, biofilms formed on SS are shown to be more susceptible to biocides than those formed on plastic materials, such as high-density polyethene (HDPE) and polystyrene (PS) [10,11]. However, plastic materials are indispensable in industrial settings due to their flexible application, (bio)corrosion resistance, and low cost. For instance, HDPE is broadly used in water systems, particularly for DWDS and food industries, offering high mechanical performance [12]. Additionally, HDPE has also been used for other purposes related to the food industry, such as the production of larger mouldings (transport and storage tanks), modular conveyor belts, sheet, tube, bearings, and gears [13,14].

Biofilms are commonly described as stratified layered structures with different tolerances to chemical and mechanical stresses [15,16]. CIP efficiency is highly improved when high shear stress is applied [4,17,18]. Therefore, the hydrodynamic effects on biofilm removal and biofilm characteristics during the cleaning step should be closely investigated. This is of particular concern when the aim is to design sustainable control strategies: it is important to assess and optimise the amount of biofilm removed for different flow conditions in order to save water and energy. Previous works reported some considerations about the influence of shear stress ($\tau_{w}$) on biofilm removal. Gomes et al. [6] combined chemical disinfection with mechanical treatment, proving that the increase of $\tau_{w}$ allowed biofilm removal from polyvinyl chloride (PVC) and demonstrated the existence of a basal layer highly tolerant to $\tau_{w}$. Ochoa et al. [19] used a Taylor-Couette reactor to study the non-uniform distribution of local $\tau_{w}$ on previous biofilms formed under low $\tau_{w}$, noticing different patterns of erosion according to the $\tau_{w}$ used. Mathieu et al. [20] assessed the hydrodynamic effects on the erosion of drinking water biofilms formed on HDPE.

The purpose of this study was to understand the effects of the quaternary ammonium compound (QAC) and benzyldimethyldodecyl ammonium chloride (BDMDAC) on the control of single- and dual-species biofilms of *Bacillus cereus* and *Pseudomonas fluorescens*, two bacteria typically encountered in biofilms found in the food industry [7,21,22,23]. The effects of BDMDAC were assessed in different stages of biofilm development: initial stages of bacterial interaction with the surface (i.e., on the alteration of bacterial surface physical and chemical properties, and the removal of adhered bacteria on HDPE surfaces) and in the removal of mature biofilms from HDPE. BDMDAC already proved to be efficient in killing *P. fluorescens* biofilms [24]. However, no information exists on its action on biofilm removal. Therefore, this work aims to fill this information gap by providing novel data on the removal of biofilms formed on HDPE by BDMDAC action. Moreover, it also aims to understand the role of the combined action of BDMDAC and mechanical treatment in biofilm removal.

## 2. Materials and Methods

### 2.1. Bacteria and Culture Conditions

*Pseudomonas fluorescens* ATCC 13525^T^ and a *Bacillus cereus* strain that were previously isolated from a disinfectant solution and identified by 16S rRNA gene sequencing [6] were used in this study. These bacteria were used as representative food spoilage microorganisms detected on food industry surfaces [21,22,23]. Bacterial cells were grown overnight in batch cultures using a concentrated nutrient medium (CNM: 5 g/L glucose, 2.5 g/L peptones and 1.25 g/L yeast extract, in phosphate buffer (PB: 0.2 M KH_2_PO_4_; 0.2 M Na_2_HPO_4_, pH 7)), at 27 ± 2 °C and under 120 rpm agitation in an orbital incubator (AGITORB 200, Aralab, Sintra, Portugal) [25]. All the components were obtained from Merck (VWR, Alfragide, Portugal).

### 2.2. Substratum

High-density polyethene (HDPE) was used as a representative substratum from industrial areas. Flat HDPE slides of 3 cm^2^ or 1 cm^2^ with thicknesses of 1.5 mm (Neves & Neves, Muro, Portugal), were used for contact-angle measurements and the initial cell adhesion assays, respectively. HDPE cylinders (length = 5.0 cm; diameter = 2.5 cm) were used for biofilm formation in the rotating cylinder reactor (RCR). The HDPE cylinders were cleaned and sterilised according to Gomes et al. [5].

### 2.3. Characterisation of Substrate and Bacterial Surfaces

The characterisation of bacterial and substrate surface was performed by the determination of surface charge (Section 2.3.1) and the hydrophobicity and surface tension parameters (Section 2.3.2) in the presence and absence of BDMDAC. The study of these characteristics allowed the determination of the physical and chemical interactions between bacteria and HDPE in the presence of BDMDAC. Such thermodynamic-based information predicted the initial stages of adhesion and the role of BDMADAC on bacteria-HDPE interaction (Section 2.4).

#### 2.3.1. Surface Charge—Zeta Potential

The electrostatic component of surface potential is commonly described by the zeta potential as a measurement of electrical surface charge. The zeta potential of bacterial suspensions (BDMDAC non-exposed and exposed) was determined in previous work [26]. The measurements of the zeta potential of HDPE were performed using a Nano Zetasizer (Malvern Instruments, UK). For that, HDPE coupons were washed and reduced to small particles (from 10 to 100 µm). The electrophoretic mobilities ($\mu_{e}$) were measured at an applied voltage of 150 V and 22 °C, then converted to zeta potential values using the Helmholtz Von Smoluchowski relation [27]. The experiments were performed in triplicate and repeated three times.

#### 2.3.2. Physicochemical Characterisation: Hydrophobicity and Surface Tension

Bacteria and substrate hydrophobicity and surface tension parameters were determined by the sessile-drop-contact-angle method as described by Busscher et al. [28] and the contact angles were determined according to Simões et al. [29], at room temperature using three different liquids: two polar—water and formamide—and one apolar—α-bromonaphtalene (Sigma, Algés, Portugal). The contact angle of HDPE was determined directly on the surface of cleaned and new coupons. To assess the contact angles of bacterial surfaces, lawns were prepared using suspensions of bacteria exposed and unexposed to BDMDAC. The surface tension of bacteria (BDMDAC non-exposed and exposed) was obtained from previous work [26]. Reference values for surface tension components of these liquids were obtained from the literature [30]. Contact angles were determined automatically using an OCA 15 Plus (DATAPHYSICS, Filderstadt, Germany) video-based optical measuring instrument, allowing image acquisition and data analysis. At least 25 determinations were performed in three independent experiments.

The degree of hydrophobicity of bacteria and substrata were also determined through contact-angle measurements, as described by Simões et al. [31]. Through the extended Young equation (Equation (1), the contact angles (θ in degrees) of a liquid (L) on a solid surface (i—bacterium or substratum) are related to total surface tension ($\gamma_{i}$, mJ/m^2^), which can be separated into two compounds ($\gamma_{i}=\gamma_{i}^{LW}+\gamma_{i}^{AB}$): Liftshitz-van der Waals ($\gamma_{i}^{LW}$) and Lewis acid-base ($\gamma_{i}^{AB}=2\sqrt{{\gamma_{i}}^{+}{\gamma_{i}}^{-}}$), where $\gamma^{+}$ and $\gamma^{-}$ are the electron-acceptor and electron-donor parameters, respectively.

(1) $$\left(1+cos\;cos\;\theta \;\right)\;\gamma_{L}\;=\;2\;\left(\sqrt{\gamma_{i}^{LW}\gamma_{L}^{LW}}+\sqrt{\gamma_{L}^{+}\gamma_{i}^{-}}+\sqrt{\gamma_{i}^{-}\times \gamma_{L}^{+}}\right)$$

The degree of hydrophobicity is expressed as the free energy of interaction between two entities of that material, when immersed in water (w)–$\Delta G_{iwi}$, mJ/m^2^ (Equation (2). If the interaction between two entities is stronger than the interaction of each entity with water $\Delta G_{iwi}<0$ mJ/m^2^, the material is considered hydrophobic. Oppositely, if $\Delta G_{iwi}>0$ mJ/m^2^, the material is hydrophilic. The hydrophobicity values of BDMDAC-untreated and -treated bacteria were previously published by Lemos et al. [26].

(2) $$\Delta G_{iwi}=-2{\left(\sqrt{\gamma_{i}^{LW}}-\sqrt{\gamma_{w}^{LW}}\right)}^{2}+4\left(\sqrt{\gamma_{i}^{+}\gamma_{w}^{-}}+\sqrt{\gamma_{i}^{-}\gamma_{w}^{+}}-\sqrt{\gamma_{i}^{+}\gamma_{i}^{-}}-\sqrt{\gamma_{w}^{+}\gamma_{w}^{-}}\right)$$

### 2.4. Free Energy of Adhesion

The obtained characterisation of HDPE and bacteria physicochemical properties allowed the assessment of the free energy of adhesion per unit area between bacteria and substratum, as described by Simões et al. [32]. When studying the interaction between two substances (1 and 2) that are immersed or dissolved in water, the total interaction energy per unit area ($\Delta G_{1w2}^{\;}$, mJ/m^2^) can be expressed as:

(3) $$\Delta G_{1w2}^{\;}=\Delta G_{Swm}^{LW}+\Delta G_{Swm}^{AB}$$

where

$$\Delta G_{1w2}^{LW}=-2\left(\sqrt{\gamma_{2}^{LW}}-\sqrt{\gamma_{w}^{LW}}\right)\left(\sqrt{\gamma_{1}^{LW}}-\sqrt{\gamma_{w}^{LW}}\right)$$

and

$$\Delta G_{1w2}^{AB}=2\left[\sqrt{\gamma_{w}^{+}}\left(\sqrt{\gamma_{1}^{-}}+\sqrt{\gamma_{2}^{-}}-\sqrt{\gamma_{w}^{-}}\right)+\sqrt{\gamma_{w}^{-}}\left(\sqrt{\gamma_{1}^{+}}+\sqrt{\gamma_{2}^{+}}-\sqrt{\gamma_{w}^{+}}\right)-\sqrt{\gamma_{1}^{-}\gamma_{2}^{+}}-\sqrt{\gamma_{1}^{+}\gamma_{2}^{-}}\right]$$

Thermodynamically, if $\Delta G_{1w2}^{\;}<0$ mJ/m^2^, adhesion is favoured, whereas if $\Delta G_{1w2}^{\;}>0$ mJ/m^2^, adhesion is not expected to occur.

### 2.5. Adhesion Assays

Bacterial adhesion to a surface is the first step for biofilm development. Therefore, flat coupons of HDPE (1 cm^2^ slide) were used to assess the effect of BDMDAC on the control of initial bacterial adhesion. The coupons were inserted vertically in the wells of 48-well microtiter plates (Nunc, Roskilde, Denmark). Each well was inoculated with a fresh sterile growth medium with 10% of bacterial suspension (at 1 × 10^8^ cells/mL), ensuring a working volume of 1.2 mL. The initial adhesion was assessed for each species individually but also for dual-species simultaneously. The study of the adhesion of dual-species aimed to simulate real industrial contamination where diverse bacterial species coexist on a surface [3,7]. The microtiter plates were incubated for 2 h in an orbital shaker (120 rpm, 27 ± 2 °C) to allow initial adhesion. Afterwards, the content of each well was discarded and the coupons were washed with sterile phosphate buffer (PB) to remove loosely attached cells. After initial bacterial adhesion and the washing step, BDMDAC treatment was performed for 30 min. For that, 1.2 mL of BDMDAC at 300 µg/mL, prepared in PB, was added to new wells where colonised coupons were inserted in. At the end of the chemical treatment, each well was washed twice with PB in order to neutralise the biocide to sub-lethal levels [33]. Control experiments were performed with PB instead of BDMDAC.

The quantification of total adhered bacteria was obtained by directly staining the cells in coupons with a drop of 4,6-diamino-2-phenylindole (DAPI) (Sigma) at 0.5 µg/mL in the dark for 5 min [34]. Total cells were visualised under an epifluorescence microscope (LEICA DMLB2) using a mercury lamp HBO/100 W/3 and the optical 359 nm excitation filter in combination with a 461 nm emission filter. The images of adhered cells were acquired with a CCD camera using IM50 software (LEICA). A total of 20 fields were counted and at least three independent coupons were used to calculate total cells per cm^2^.

### 2.6. Biofilm Formation–Experimental Set-Up

**Figure 1:**
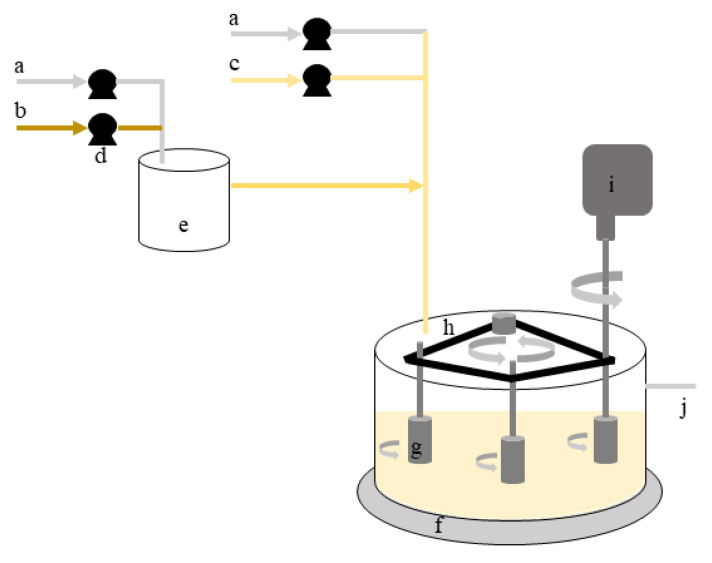
Scheme representative of the rotating cylinder reactor: (**a**)—inlet of sterile air; (**b**)—inlet of concentrated nutrient medium; (**c**)—inlet of diluted nutrient medium; (**d**)—peristaltic pumps; (**e**)—chemostat; (**f**)—rotating cylinder reactor; (**g**)—testing cylinders (samplers); (**h**)—synchronising belt that connect the three cylinders; (**i**)—stirrer that controls rotation speed; (**j**)—outlet of bacterial suspension.

Single- and dual-species biofilms of *B. cereus* and *P. fluorescens* were formed in the rotating cylinder reactor (RCR) for 7 days, as previously described by Lemos et al. [26,35]. Briefly, the RCR operated in steady-state with three cylinders of HDPE rotating simultaneously. This system consisted of a main bioreactor (RCR) and a chemostat as presented in Figure 1.

The main bioreactor consists of a tank (external diameter = 28 cm; height = 16 cm; thickness = 0.4 cm) with 5 L of operating volume and 3 L left for aeration. The cylinders were vertically placed in a triangular configuration, distanced from each other by 12 cm and around 6 cm distanced to the tank wall. This configuration allowed the reduction of hydrodynamic influences between different cylinders and/or tank walls. An overhead stirrer (IKA-Werke GmbH & Co. KG, Staufen, Germany) with a speed range from 50 to 2000 rpm provided the simultaneous rotation of cylinders as they were connected by a synchronous belt. Adjusting the rotating velocity, it is possible to control the hydrodynamic conditions for biofilm formation. The rotating velocity is related to the Reynolds number of agitation ($Re_{A}$) accordingly to Equation (4) [36], where $D_{A}^{\;}$ (m) is the diameter of the cylinder, N (s^−1^) is rotation speed, and ρ (kg/m^3^) and μ (kg/m.s) are fluid density and viscosity, respectively.

(4) $$Re_{A}=\frac{D_{A}^{2}N\rho }{\mu }$$

For a Newtonian fluid, the Fanning friction factor (f) establishes the correlation between $\tau_{w}$ and the velocity of the flow kinetic energy defined by Perry and Green [37], according to Equation (5) with v (m/s) as the flow velocity. For this study, the relationship between f and $Re_{A}$ for a rotating electrode under turbulent flow conditions (the critical $Re_{A}$ is 200) by Gabe and Walsh [38] was applied Equation (6). Table 1 summarises the estimated values of $Re_{A}$ and $\tau_{w}$ at each rotation speed used in this work.

(5) $$f=\frac{2\tau_{w}}{\rho v^{2}}$$

(6) $$f=0.158Re_{A}^{-0.3}$$

The RCR was fed with 0.8 L/h of sterile diluted nutrient medium (DNM: 1:100 dilution of CNM). The RCR was also continuously supplied with 10 mL/h of a planktonic culture of *B. cereus* and/or *P. fluorescens* in the exponential phase of growth from one or two chemostats of 0.5 L. The chemostat was continuously aerated, agitated, and fed with 10 mL/h of CNM. The aeration was provided via a cellulose acetate syringe filter with a pore size of 0.22 µm (Whatman, VWR, Portugal) to the RCR and chemostat. The mature biofilms were allowed to grow for 7 days at 27 ± 2 °C [39]. Three independent biofilm formation experiments were performed for each condition studied.

The number of spores of *B. cereus* in single- and dual-species-adhered cells and biofilms was assessed by surface plating (300 mL sample) after heat treatment (80 °C, 5 min) [7,16].

### 2.7. Biofilm Characterization

After biofilm formation in the RCR, cylinders containing biofilms were carefully inserted into a 250 mL beaker containing 200 mL of PB to remove non-adhered and/or weakly adhered bacteria. Then, the single- and dual-species biofilms were characterised in terms of thickness, wet and dry mass, cell density, and EPS content. Wet biofilm mass was obtained by subtracting the weight of the cylinder without biofilm (before the experiment) to the weight of the cylinder with biofilm (after the washing step), as described by Gomes et al. [6], Lemos et al. [26,35], and Simões et al. [39]. Biofilm thickness was determined using a digital micrometre (VS-30 H, Mitsubishi Kasei Corporation, Yokohama, Japan) according to Teodósio et al. [40].

Afterwards, for the following characterisation, biofilm was scrapped from the cylinder surface with a stainless steel scrapper. Then, scrapped biofilms were resuspended in 10 mL of sterile extraction buffer (0.76 g/L Na_3_PO_4_·H_2_O, 0.36 g/L Na_2_HPO_4_·H_2_O, 0.53 g/L NaCl, 0.08 g/L KCl), and homogenised by vortexing for 30 s at maximum power input [7].

For the quantification of total cell density, a 500 µL aliquot of the homogenised biofilm suspension was filtered through a 0.22 µm Nucleopore^®^ (Whatman, Middlesex, UK) black polycarbonate membrane. The membrane was stained with DAPI and left in the dark for 5 min [34]. After incubation, the total cell counts were assessed by epifluorescence microscopy (LEICA DMLB2).

The EPS characterisation was performed in order to understand the main composition of each biofilm tested in order to understand the role of EPS in biofilm removal by chemical and mechanical treatments. The EPS extraction was performed according to a previously described method [41]. Briefly, Dowex^®^ Marathon© resin (NA^+^ form, strongly acidic, 20–50 mesh, Sigma) was added to the biofilm suspension (1 g per 10 mL of biofilm suspension). The extraction took place for 4 h at 400 rpm and 4 °C, using always identical 25 mL beakers. The extracellular components present in the supernatant were separated from cells through centrifugation (3700× *g*, 5 min). Afterwards, the total and extracellular biofilm protein quantification was performed as described by Lowry et al. [42], modified by Peterson [43], using the Total Protein Kit, Micro Lowry, Peterson’s Modification (Sigma, Algés, Portugal), with bovine serum albumin as standard. The total and extracellular polysaccharides were quantified through the phenol-sulphuric acid method of DuBois et al. [44] using glucose as standard. The homogenised biofilm suspensions were placed in a furnace at 550 °C for 2 h to determine the total volatile solids (TVS) of the sample as the biofilm dry mass [45]. The water content was estimated as the difference between the wet mass and the dry mass of biofilms. The biofilm density was calculated as the ratio between the dry biofilm mass and its volume (estimated as the product of biofilm thickness and adhesion surface area) [46].

### 2.8. Chemical Treatment

A cationic surfactant solution of BDMDAC (Sigma-Aldrich) was used for the chemical treatment of biofilms. Solutions with a concentration of 300 µg/mL were prepared in PB. After 7 days of biofilm formation, the HDPE cylinders with biofilms were removed from the RCR and washed in sterile PB to remove weakly or non-adherent bacteria. Then, they were immersed in 250 mL glass beakers (diameter: 6.8 cm) containing 200 mL of a BDMDAC solution. The chemical treatment was carried out for 30 min under the $\tau_{w}$ used for biofilm formation (0.70 Pa). The control (untreated biofilm) was carried out with PB instead of BDMDAC. After chemical treatment, a neutralisation step was performed to dilute the biocide to residual levels, as described by Johnston et al. [31]. The wet weight of the cylinders plus biofilm attached was determined before ($X_{biofilm}$) and after chemical treatment (X) [6,26,35,39]. The wet biofilm mass that was removed from the surface area of each cylinder was expressed in terms of the percentage of biofilm removal (Equation (7)):

(7) $$Biofilm\;removal\;\left(\%\right)=\frac{X_{biofilm}-X_{\;}}{X_{biofilm}}\times 100$$

Afterwards, treated biofilm was scrapped and resuspended in 10 mL of sterile PB for the determination of biofilm cellular density, as described in Section 2.8.

### 2.9. Mechanical Treatment

The biofilm removal by hydrodynamic stress was assessed according to the method described by Simões et al. [39]. Colonised cylinders were immersed in 200 mL of PB and submitted to 30 s pulse of increasing $\tau_{w}$ (from 0.7 to 17.7 Pa). The wet weight of the cylinders plus biofilm attached was determined before and after exposure to hydrodynamic stress in order to determine the biomass removal after each pulse, according to Equation (7). The same procedure was followed with the control assay, i.e., with the cylinder plus biofilm non-exposed to BDMDAC. The amount of biofilm that remained adhered after exposure to the complete series of $\tau_{w}$ was expressed as the percentage of biofilm remaining ($X_{remaining}$) on the cylinder surface after treatments, according to Equation (8), where $X_{biofilm}$ is the wet mass of the non-exposed biofilm [6,26,35,39]:

(8) $$Biofilm\;remaining\;\left(\%\right)=\frac{X_{remaining}}{X_{biofilm}}\times 100$$

### 2.10. Statistical Analysis

The data were analysed using the statistical program SPSS version 21.0 (Statistical Package for the Social Sciences). The mean and standard deviation (SD) within samples were calculated for all cases. At least three independent experiments were performed for each condition tested. All data were analysed by the application of the non-parametric Kruskal–Wallis test (confidence level ≥ 95%).

## 3. Results and Discussion

HDPE is a surface material widely used in industrial processes and equipment, whose relationship to disinfection efficiency remains to be understood. In this study, the thermodynamic approach was applied to assess the interaction between bacteria and HDPE, allowing the prediction of the initial adhesion process. This approach further allows understanding of whether BDMDAC may have specific effects on the control of a monolayer of adhered cells. According to Lemos et al. [26], both *P. fluorescens* and *B. cereus* (BDMDAC-untreated) were hydrophilic ($\Delta G_{iwi}=$ 14.8 and 29.5 mJ/m^2^, respectively). The BDMDAC treatment caused the increase of hydrophilicity of *P. fluorescens*, but no difference was observed for *B. cereus*. The potential of bacteria to adhere to HDPE was thermodynamically predicted by the quantification of the free energy of adhesion per area unit between the bacteria and HPDE (Table 2). The adhesion potential of *B. cereus* was thermodynamically more favourable than that of *P. fluorescens*. Thus, the lowest degree of hydrophilicity of *B. cereus* allowed the higher thermodynamic propensity to adhere to HDPE (a hydrophobic substrata) as obtained by [31]. The effect of BDMDAC treatment was controversial since it decreased the potential of *B. cereus* adhesion and favoured that of *P. fluorescens.* However, the potential of *P. fluorescens* to adhere to HDPE in the presence of BDMDAC remained lower than for *B. cereus*. Lemos et al. [26] also performed a similar study regarding the prediction of *B. cereus* and *P. fluorescens* adhesion to SS and PMMA, in the presence and absence of BDMDAC. A comparative analysis between the present results and those from Lemos et al. [26] reveals that the adhesion potential of both bacteria was thermodynamically more favourable on PMMA followed by HDPE and SS, both after and before BDMDAC exposure.

The initial adhesion process is related to the hydrophobic properties of the bacteria and substratum in addition to the electrostatic interactions established between them [47,48]. For this reason, the surface charge of bacteria was also determined. Both bacteria are negatively charged and BDMDAC exposure increased charge to less-negative values (−15.6 mV for *B. cereus* and −4.1 mV for *P. fluorescens*) [26]. This effect was also observed by Ferreira et al. [24] when exposing a *P. fluorescens* strain isolated from drinking water to that biocide, an effect apparently related to the cationic nature of BDMDAC.

**Figure 2:**
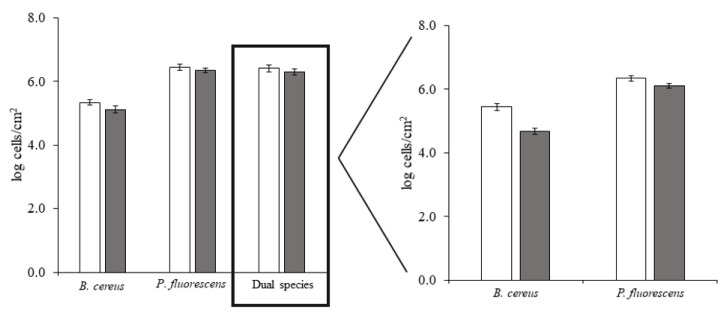
Number of *B. cereus* and *P. fluorescens* in single- and dual-species adhered on HDPE surface, before (□) and after (■) BDMDAC treatment. Mean values ± SDs for at least three replicates are illustrated.

*In vitro* adhesion assays were performed to assess bacterial adhesion on HDPE and the effects of BDMDAC on initial adhesion control. These assays are complementary to the previous thermodynamic-based analysis, highlighting the role of biological aspects. The results demonstrated that both bacteria in single- and dual-culture adhered to HDPE (Figure 2). *B. cereus* adhered at a lower extent (5.3 log cells/cm^2^) than *P. fluorescens* (6.4 log cells/cm^2^) and the dual culture (6.4 log cells/cm^2^). Thus, conversely to the thermodynamic approach, *B. cereus* had lower adhesion potential on HDPE than *P. fluorescens* (dominant species) for single- and dual-species cultures. The levels of *P. fluorescens* adhesion on HDPE are similar to those observed on PMMA and SS, while *B. cereus* adhered at a higher extent on SS and PMMA [26]. The BDMDAC treatment caused a modest decrease in the number of adhered bacteria: 0.1 log cells/cm^2^ (dual culture and *P. fluorescens*) and 0.2 log cells/cm^2^ (*B. cereus*).

In general, the results from the thermodynamic approach did not fit those obtained with the in vitro adhesion assays. This is usually attributed to the existence of other mechanisms involved in the initial adhesion rather than just hydrophobic and electrostatic interactions. The extracellular appendages and proteins, such as pili, flagella, fimbriae, and outer membrane proteins play an important role in cellular motility and attachment [49,50]. Other factors also have an influence on bacterial adhesion including the substratum surface properties (surface charge and roughness) and environmental process conditions (temperature, pH, bacterial concentration, time of contact, chemical treatment, or the fluid flow conditions) [51,52,53].

Mature biofilms were formed on HDPE surfaces and their characterisation is depicted in Table 3. The characterisation of biofilms may be of utmost importance in the interpretation of removal results after mechanical and chemical treatments.

Dual-species biofilms were the thickest, followed by *B. cereus* and *P. fluorescens* biofilms (*p* < 0.05). However, the biofilm density of *P. fluorescens* biofilms was higher (13.1 mg/cm^3^) than that of *B. cereus* biofilms and dual-species biofilms, which had similar values: 3.6 and 3.7 mg/cm^3^, respectively (*p* < 0.05). These differences can be explained by a more cohesive biofilm (thinner) with the same cell density [25]. In fact, the cell density was similar for the three biofilms (6.67–6.94 log cells/cm^2^) (*p* > 0.05). *P. fluorescens* biofilms had the highest productivity in terms of dry mass, followed by the dual-species biofilms (*p* < 0.05). Statistically significant differences were observed in terms of polysaccharides and proteins composition, in both total and extracellular concentrations (*p* < 0.05). The production of extracellular polysaccharides was favoured for *B. cereus* and *P. fluorescens* biofilms, whereas dual-species biofilms produced more extracellular proteins (*p <* 0.05). According to previous results obtained by Lemos et al. [26] and Lemos et al. [35] using SS and PMMA in the RCR, biofilms showed different characteristics, explained by differences in the physicochemical properties of bacteria and the substrata, which resulted in different surface–surface interactions. Moreover, the number of spores of *B. cereus* was determined in order to provide information on their density in the total population (spores and vegetative cells). In single- and dual-species biofilm tests, *B. cereus* spores were never detected at a density > 1 spore per million vegetative cells. This is apparently due to the continuous operating conditions under which biofilms were formed, in addition to the culture conditions used that favoured vegetative cell growth [7]. The dual-species biofilms on HDPE were predominantly colonised by *B. cereus* (approximately 82%). This was inconsistent with the results from in vitro adhesion assays, emphasising that the first stages of biofilm formation may not reflect the characteristics of the mature biofilm. This highlights the need to evaluate biofilm-control strategies focusing on different development stages. In fact, the understanding of the relationship between adhesion and biofilm formation can be useful to know the role played by particular microorganisms in the system and to develop reliable and effective control strategies in the early stages of biofilm formation. However, it is important to reinforce that the initial stages do not correspond to the characteristics and behaviour of mature biofilms. The differences between the platforms used in initial adhesion assays (48-wells microtiter plates) and the study of mature biofilms (RCR), may also justify the differences observed, mainly due to the distinct hydrodynamic conditions used.

**Figure 3:**
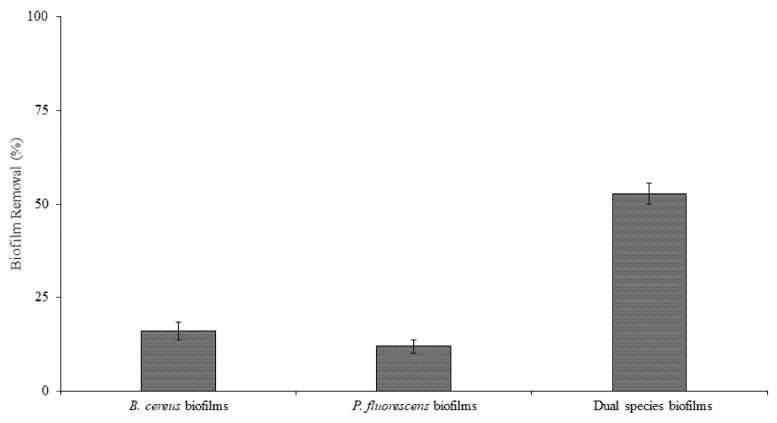
Biofilm removal after submitting the *B. cereus*, *P. fluorescens,* and dual-species biofilms to BDMDAC at 300 µg/mL under 0.04 Pa. Mean values ± SDs for at least three replicates are given.

The single- and dual-species mature biofilms were exposed to BDMDAC under the same hydrodynamic conditions used for biofilm formation (0.70 Pa), and the effect of the chemical treatment on biofilm removal was assessed (Figure 3). *P. fluorescens* biofilms were the most tolerant to BDMDAC exposure (only 12% of the biofilm removed by BDMDAC action) (*p* < 0.05), which can be apparently explained by the higher amount of EPS and higher density when compared to the other biofilms. The EPS matrix is related to mass transfer limitations, and its components may interact with the biocide, reducing their availability to act against the biofilm cells [54,55,56]. The dual-species biofilms were significantly more susceptible to the BDMDAC treatment (53% biofilm removal) than the single-species biofilms (16% of *B. cereus* and 12% of *P. fluorescens* biofilms) (*p* < 0.05). So, on HDPE, this particular species association increased the biocidal susceptibility to removal. However, the BDMDAC treatment was not effective to completely remove biofilms. In fact, the chemical treatments alone have already proved to be insufficient [39,57]. Thus, the residual microorganisms after chemical treatment can promote the rapid re-establishment/regrowth of biofilms, usually showing a decreased susceptibility to subsequent chemical treatments [58].

**Figure 4:**
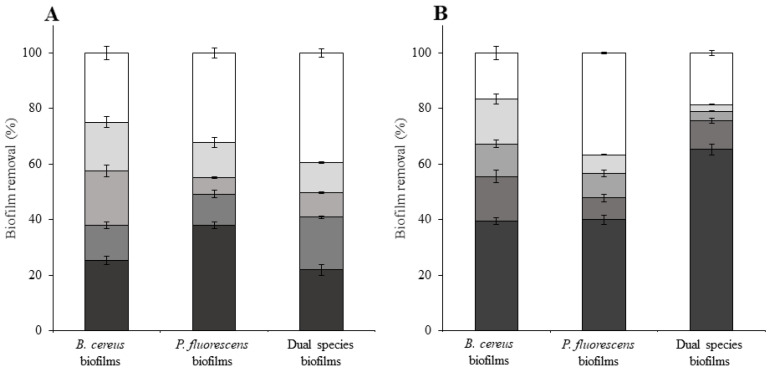
Biofilm removal caused by the increasing series of shear stress (mechanical treatment) on chemically (BDMDAC) untreated (**A**) and treated (**B**) biofilms). ■—exposure to 1.66 Pa; ■—exposure to 5.50 Pa; ■—exposure to 10.9 Pa; ■—exposure to 17.7 Pa. The white bar (□) represents the amount of biofilm remaining after the mechanical treatment. Mean values ± SDs for at least three replicates are given.

To achieve a more effective biofilm removal, the remaining biofilms from chemical treatments were subjected to a sequential increase in shear stress (Figure 3). The $\tau_{w}=$ 1.66 Pa (the first shear stress value applied in the series) caused the most significant biofilm removal. However, the first step of mechanical stress did not cause such removal of chemically non-treated biofilms of *B. cereus* and dual-species biofilms, as observed for *P. fluorescens* biofilms (*p* < 0.05) (Figure 4A). On the other hand, dual-species biofilms treated with BDMDAC were more susceptible to the lower shear stress than BDMDAC-treated, single-species biofilms. The $\tau_{w}=$ 5.5 Pa did not remove as much biofilm as the first one, indicating that there was a possible compression of the structure. Paul et al. [15] also verified that during erosion tests, detachment occurred under $\tau_{w}$ > 2 Pa. However, above that value, compression mechanisms influenced more the physical biofilm stability. As biofilms became progressively compressed by the $\tau_{w}$ forces, the biofilm porosity changed, and mechanical stability and detachment processes were also affected. According to Hornemann et al. [59], the QACs effects on EPS induced the elimination of the diffusive restrictions of biomacromolecules in the biofilm. These mechanisms suggest that some physical alterations also occurred, explaining the higher removal obtained for the BDMDAC-treated biofilms when exposed to the higher $\tau_{w}$.

The complete series of $\tau_{w}$ was not enough to completely remove BDMDAC-untreated and treated biofilms. Even after mechanical treatment, there was still 16%, 36%, and 19% of BDMDAC-treated biofilm mass covering the HDPE surface, for *B. cereus, P. fluorescens,* and the dual-species biofilms, respectively. In fact, the chemical treatment improved the impact of mechanical treatment for *B. cereus* and dual-species biofilms, reducing the percentage of remaining biofilm on the HDPE surface from 25% and 40% to 17% and 19%, respectively (*p* < 0.05). For *P. fluorescens* biofilms, the previous chemical treatment was apparently indifferent in reducing its mechanical stability (*p* > 0.05). Pechaud et al. [60] used a combination of mechanical and enzymatic treatments to control multispecies biofilms and observed that hydrodynamic treatments caused both biofilm detachment and compaction. The enzymatic treatments applied alone were not effective in biofilm removal, but the combination of treatments resulted in up to 90% biomass removal. These results propose that a universal biofilm control strategy, valid for a broad range of microbial species, can hardly be achieved. Additionally, the use of HDPE as an industrial surface showed similar results on biofilm remaining when the combination of treatments (BDMDAC and hydrodynamic stress) was applied to biofilms formed on SS [26]. Moreover, the biofilm remaining on HDPE surfaces after combined treatments were lower than that reported by Lemos et al. [26] for biofilms formed on PMMA. However, the dual-species biofilms formed on HDPE were more susceptible to BDMDAC action than the biofilms formed on SS and PMMA [26]. It is important to consider that the type of surface accounts for the antimicrobial effectiveness. For instance, Poimenidou et al. [11] verified that biofilms formed on polystyrene had higher cell density and more tolerance to disinfectants (peracetic acid and QACs) than those formed on SS. Moreover, the type of surface also accounts for the biofilm mechanical stability, as the susceptibility of biofilms to increasing shear stresses can be different for biofilms formed on different surfaces. For instance, biofilms formed on HDPE were generally less susceptible to hydrodynamics than biofilms formed on PMMA (except for dual-species biofilms that had a similar susceptibility to mechanical treatments on both materials) [26]. Moreover, *P. fluorescens* biofilms and dual-species biofilms formed on SS [26] were more susceptible to hydrodynamic conditions than their counterparts formed on HDPE (*B. cereus* biofilms formed on HDPE and SS have similar susceptibility to mechanical treatment) [26].

## 4. Conclusions

The RCR proved to be a versatile tool to investigate the efficacy of a combined strategy of chemical and mechanical treatment of single- and dual-species biofilms of *B. cereus* and *P. fluorescens* formed on HDPE. Predictions of microbial adhesion provided by the thermodynamic approach failed to confirm the in vitro assays, which indicated that *P. fluorescens* adhered in higher numbers than *B. cereus*. BDMDAC treatment decreased the number of cells adhered more extensively for *B. cereus*. However, the results highlight that the initial adhesion may not reflect the characteristics of mature biofilms. Single- and dual-species biofilms of *B. cereus* and *P. fluorescens* on HDPE were distinct, with different phenotypic characteristics and behaviour to chemical and mechanical stresses. The treatment of 7-days-old single- and dual-species biofilms with BDMDAC was inefficient on the total biofilm control. *P. fluorescens* biofilms were the most tolerant to the biocide and those formed by both species were the most susceptible. The application of distinct $\tau_{w}\;$ emphasized the inherent mechanical stability of single- and-dual species biofilms. Low $\tau_{w}$ values (1.56 Pa) caused higher erosion of the biofilm whereas the higher $\tau_{w}\;$ values seem to cause a compression indicated by lower removal percentages. The combination of BDMDAC with mechanical treatment by increasing $\tau_{w}\;$ enhanced the biofilm removal for *B. cereus* and dual-species biofilms. However, even with the synergistic chemical and mechanical treatment, total biofilm control was not achieved for single- and dual-species biofilms formed on HDPE. Despite that, attending the information available on literature [26], biofilms formed on HDPE had similar susceptibility to combined treatments as those formed on SS and were more susceptible than those formed on PMMA. The results obtained demonstrated that the mechanical stability of biofilms as well as their susceptibility to BDMDAC is influenced by the type of surface/material.

## Figures and Tables

**Table 1:** Estimated values of $Re_{A}$ and *τ_w_* at the rotation speeds (*N*) applied in this study according to Equations (4)–(6).

N (s^−1^)	$Re_{A}$	$\tau_{w}\;(\mathrm{Pa})$
3.84	2400	0.70
6.40	4000	1.66
13.0	8100	5.50
19.4	12,100	10.9
25.8	16,000	17.7

**Table 2:** Free energy of adhesion ($\Delta G_{1w2}$ in mJ/m^2^) between BDMDAC-untreated (control) and -treated bacteria, and HDPE.

	*B. cereus*		*P. fluorescens*	
	Control	BDMDAC	Control	BDMDAC
HDPE	−13.1	−6.5	7.8	−5.8

**Table 3:** Characteristics of *B. cereus* and *P. fluorescens* single- and dual-species biofilms on HDPE, before chemical or mechanical treatments. Mean values ± SDs for at least three replicates are given.

	*B. cereus*	*P. fluorescens*	Dual Species
Thickness (µm)	526 ± 8	278 ± 71	880 ± 90
Dry mass (mg/cm^2^)	0.191 ± 0.02	0.365 ± 0.07	0.324 ± 0.09
Volumetric density (mg/cm^3^)	3.6 ± 0.3	13.1 ± 1.9	3.7 ± 1.1
Cellular density (log cells/cm^2^)	6.67 ± 0.11	6.94 ± 0.20	6.90 ± 0.04 *
Water content (% of total biofilm mass)	97.9 ± 0.7	95.0 ± 3.1	99.3 ± 2.5
Extracellular polysaccharides (% of total biofilm polysaccharides)	72.1 ± 1.5	69.8 ± 2.1	59.4 ± 0.7
Extracellular proteins (% of total biofilm proteins)	50.9 ± 0.5	34.3 ± 0.6	72.0 ± 4.5

* 6.75 (±0.03)/81.9% of *B. cereus* and 6.3 (±0.2)/18.1% of *P. fluorescens*.

## Data Availability

The data that support the findings of this study are available from the corresponding author upon reasonable request.
